# GORetriever: reranking protein-description-based GO candidates by literature-driven deep information retrieval for protein function annotation

**DOI:** 10.1093/bioinformatics/btae401

**Published:** 2024-09-04

**Authors:** Huiying Yan, Shaojun Wang, Hancheng Liu, Hiroshi Mamitsuka, Shanfeng Zhu

**Affiliations:** Institute of Science and Technology for Brain-Inspired Intelligence and MOE Frontiers Center for Brain Science, Fudan University, Shanghai 200433, China; Institute of Science and Technology for Brain-Inspired Intelligence and MOE Frontiers Center for Brain Science, Fudan University, Shanghai 200433, China; Institute of Science and Technology for Brain-Inspired Intelligence and MOE Frontiers Center for Brain Science, Fudan University, Shanghai 200433, China; Bioinformatics Center, Institute for Chemical Research, Kyoto University, Uji, Kyoto Prefecture 611-0011, Japan; Department of Computer Science, Aalto University, Espoo 00076, Finland; Institute of Science and Technology for Brain-Inspired Intelligence and MOE Frontiers Center for Brain Science, Fudan University, Shanghai 200433, China; Key Laboratory of Computational Neuroscience and Brain-Inspired Intelligence (Fudan University), Ministry of Education, Shanghai, 200433, China; Shanghai Key Lab of Intelligent Information Processing and Shanghai Institute of Artificial Intelligence Algorithm, Fudan University, Shanghai, 200433, China; Zhangjiang Fudan International Innovation Center, Shanghai, 200433, China

## Abstract

**Summary:**

The vast majority of proteins still lack experimentally validated functional annotations, which highlights the importance of developing high-performance automated protein function prediction/annotation (AFP) methods. While existing approaches focus on protein sequences, networks, and structural data, textual information related to proteins has been overlooked. However, roughly 82% of SwissProt proteins already possess literature information that experts have annotated. To efficiently and effectively use literature information, we present GORetriever, a two-stage deep information retrieval-based method for AFP. Given a target protein, in the first stage, candidate Gene Ontology (GO) terms are retrieved by using annotated proteins with similar descriptions. In the second stage, the GO terms are reranked based on semantic matching between the GO definitions and textual information (literature and protein description) of the target protein. Extensive experiments over benchmark datasets demonstrate the remarkable effectiveness of GORetriever in enhancing the AFP performance. Note that GORetriever is the key component of GOCurator, which has achieved first place in the latest critical assessment of protein function annotation (CAFA5: over 1600 teams participated), held in 2023–2024.

**Availability and implementation:**

GORetriever is publicly available at https://github.com/ZhuLab-Fudan/GORetriever.

## 1 Introduction

Understanding the functions of proteins plays a crucial role in biomedical research and is essential for comprehending life processes, disease mechanisms, and drug development. To systematically describe the functions of proteins, Gene Ontology (GO) has been developed. GO has three branches, i.e. Molecular Function Ontology (MFO), Biological Process Ontology (BPO), and Cellular Component Ontology (CCO), with over 50 000 terms (Ashburner *et al.* 2000). However, due to the high cost of biochemical experiments, only <0.1% of more than 250 million proteins collected in UniProt (The UniProt Consortium 2023), the current largest protein database, have experimental functional annotations. Therefore, it is imperative to develop high-performance computational methods for automated function prediction/annotation (AFP).

AFP is typically achieved by associating the proper GO terms with a target protein. This process can be regarded as a multi-label classification task. Many methods have been proposed to tackle AFP by using different types of data sources (Makrodimitris *et al.* 2020). Most methods concentrate on sequence information, including sequence alignment, domains, family, motifs (Marchler-Bauer *et al.* 2015, Sillitoe *et al.* 2015), and sequence-based features obtained by deep learning (Kulmanov and Hoehndorf 2020, 2022, Cao and Shen 2021). Protein language models extract deep semantic information from protein sequences by pre-training (Rao *et al.* 2019, Rives *et al.* 2021), which have achieved competitive performance on AFP (Zhu *et al.* 2022, Wang *et al.* 2023). In addition, protein–protein interactions (PPIs) (You *et al.* 2021) and protein 3D structures (Gligorijević *et al.* 2021, Lai and Xu 2021, Boadu *et al.* 2023) are also broadly used for AFP. Moreover, as demonstrated in a recent critical assessment of protein function annotation (CAFA) (Radivojac *et al.* 2013, Jiang *et al.* 2016, Zhou *et al.* 2019), the state-of-the-art AFP methods, such as GOLabeler and NetGO (You *et al.* 2018b, 2019, Yao *et al.* 2021, Wang *et al.* 2023), were achieved by ensemble methods that integrate different types of data sources.

To further advance AFP, we focus on textual information related to proteins, such as description in UniProt and expert-curated biomedical literature in SwissProt, since this information has not been considered extensively despite its usefulness for AFP. Protein functions are typically annotated by human curators. Despite the existence of relevant literature, a significant portion of proteins still awaits expert annotation using the literature review. Specifically, over 80% of proteins (around 470 000) in SwissProt have expert annotated literature, whereas merely approximately 16% (around 73 900) have experimental functional annotations (The UniProt Consortium 2023). Therefore, developing an efficient AFP method is important to mitigate this gap and save the time and cost of human curators.

Currently, very few methods use textual information for AFP. A notable example is DeepText2GO, which uses expert annotated biomedical literature to improve the prediction performance (You *et al.* 2018a). DeepText2GO generates text representations by concatenating Document to Vector (D2V) (Le and Mikolov 2014) and Term Frequency-Inverse Document Frequency (TFIDF), and then trains a classifier for each GO term (You *et al.* 2018a). A significant drawback of DeepText2GO is that the text representations based on D2V and TFIDF cannot well capture contextual information (Minaee *et al.* 2021).

In addition, ProTranslator (Xu and Wang 2022) and ProtST (Xu *et al.* 2023) showed the effectiveness of protein textual descriptions, where many of them were generated by ProtNLM (Gane *et al.* 2022) with high efficiency. Specifically, ProtST proposes a multi-modal framework to enhance the protein language model by protein descriptions for multiple downstream tasks. On the other hand, ProTranslator embeds proteins based on the protein sequence, description, and network, which puts the focus on annotating proteins with novel GO terms (zero-shot protein function prediction).

Both ProtST and ProTranslator use PubMedBERT (Gu *et al.* 2021) to encode protein descriptions but ignore the abundant information in the annotated literature, which limits their performance for AFP. In light of the above, there are three challenging issues in using literature data for better AFP: (i) how to construct an effective protein representation using annotated literature, especially when the number of annotated literature for a target protein is large; (ii) how to conduct semantic matching between proteins (descriptions, literature) and GO terms (definitions); and (iii) how to make the whole procedure feasible for the huge number of all possible protein-GO pairs.

**Figure 1.**
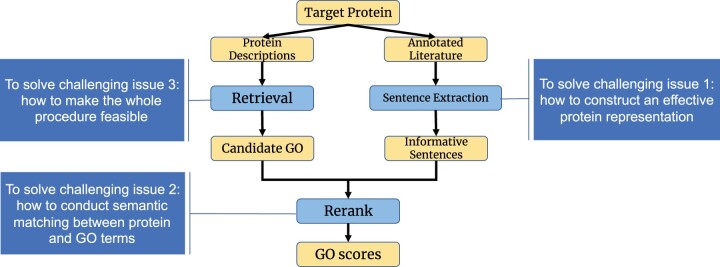
The workflow of GORetriever, with three components: Retrieval, Sentence Extraction, and Rerank. Retrieval and Sentence Extraction can be processed in parallel to generate candidate GO terms and informative sentences, respectively, for Rerank.

To address the above challenges, we propose GORetriever (Fig. 1), a two-stage framework using a deep information retrieval (IR) model with three components for high-performance AFP. To the best of our knowledge, this is the first deep-learning-based IR framework for AFP. In GORetriever, the target protein and GO terms are regarded as a query and documents, respectively: AFP is a problem of finding relevant GO terms (documents), given a target protein (query). In the first stage, an initial small set of candidate GO terms is retrieved by the **Retrieval** component from annotated proteins in the training data, which have similar descriptions to those of a target protein. At the same time, to construct effective protein representation and reduce noise, the **Sentence Extraction** component extracts the most informative sentences from the annotated literature for each protein. In the second stage, the **Rerank** component re-scores the result of the first stage, which prioritizes the most relevant GO terms by deep semantic matching of proteins and GO terms using their textual information. We note that the candidate GO terms in the first stage can be generated from another AFP method using different data sources, such as BLAST-KNN (Altschul *et al.* 1997) (sequence alignment), and LR-ESM (protein language model), which are two major components of NetGO3.0 (Wang *et al.* 2023). We validated the effectiveness of GORetriever on a large-scale dataset and observed a significant improvement compared to the state-of-the-art methods using a single source. Finally, note that incorporating GORetriever into the “learning to rank” framework of NetGO led to a significant achievement already: first place out of over 1600 teams in CAFA5 (Friedberg *et al.* 2023).

## 2 Materials and methods

### 2.1 Overview

We formulate AFP as a problem of ranking documents relevant (semantically matching) to a query, where a query denotes a target protein and documents correspond to GO terms. Specifically, we represent the textual feature of a target protein as pair x=(p,L), where *p* encapsulates the descriptions in UniProt, including recommended names $p_{\text{name}}$, species $p_{\text{species}}$, entry name $p_{\text{entry}}$, and so on, *L* denotes annotated literature in SwissProt. To characterize GO terms, we leverage GO definitions as candidate documents. The problem can be formally defined as follows: Given protein feature x=(p,L) and the whole set of GO terms $G=\{g_{1},g_{2},\ldots ,g_{n}\}$ with their respective definitions $D=\{d_{1},d_{2},\ldots ,d_{n}\}$, retrieve the most relevant subset $G^{\prime }$ to *x*, out of **G**.

**Figure 2.**
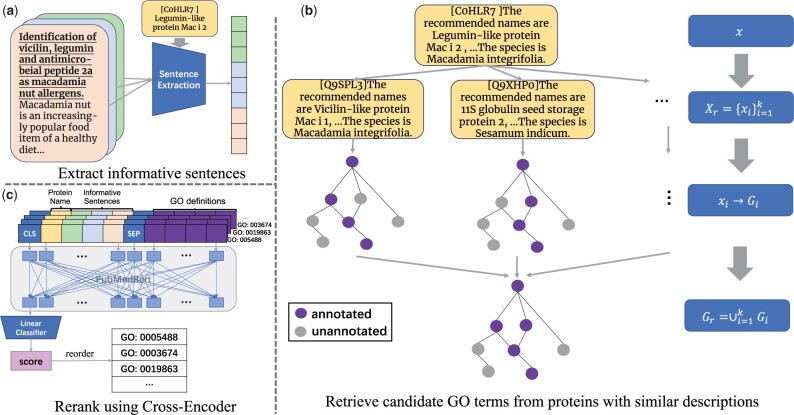
Three components of GORetriever. (a) Sentence Extraction from annotated literature. Sentences are colored to represent different literature resources, where the number of sentences for each color can be changed. (b) Retrieve candidate GO terms from proteins with similar descriptions. Similar proteins *X_r_* are first obtained and then annotated GO terms of these proteins are added to candidate set *G_r_*. (c) Rerank candidate GO terms, according to Cross-Encoder (CE). Each input of CE is a concatenation of “protein name,” “informative sentences” (from a) and “GO definitions” (from b). Candidate GO terms are reordered by the output of CE.

Figure 2 shows the three major components of GORetriever. First, due to the fact that many sentences in the annotated literature are not related to protein functions, we introduce the **Sentence Extraction** component to extract informative sentences from the literature, minimizing extraneous noise and reducing cost. Secondly, we obtain a small set of candidate GO terms based on the annotated proteins with similar descriptions through the **Retrieval** component. Finally, the **Rerank** component prioritizes the most relevant GO terms using the textual interaction of extracted protein annotations and GO terms with a deep learning model.

### 2.2 Sentence extraction: extract the most informative sentences from annotated literature

Before implementing our retrieval model, for each target protein, we collect annotated literature data *L* from SwissProt, which gives the PubMed identifiers under protein entries (Boutet *et al.* 2016), and focus on the “Title” and “Abstract” fields only. As many sentences within the annotated literature are unnecessary for protein function annotation, the **Sentence Extraction** component generates effective protein representations as well as reduces noise and computational cost.

As shown in Fig. 2a, extracting all sentences from the annotated literature and mixing them, the goal of the Sentence Extraction component is to select the most informative sentences $L(b_{i})$ for each GO branch *b_i_*. To achieve this, we score the sentences with a pre-trained Seq2seq ranking model without any additional training (Nogueira *et al.* 2020). The input is defined as:


Query: q Sentence: s Relevance: [token]

where q is “What is the [branch description] of protein [protein]?,” and “[branch description]” can be replaced by “Molecular Function” (MF), “Biological Process” (BP) or “Cellular Component” (CC), and for “[protein],” we can choose only the recommended protein names in SwissProt to avoid noise. Then we compute the relevance score for each sentence *s* in the annotated literature *L* by computing the log-likelihood probability of generating “true” in the next [token]. Finally, we reorder all sentences with respect to their corresponding probabilities and obtain a part of top-scoring sentences as the extracted context $L(b_{i})$ (see Section 3.3 for more detail).

### 2.3 Retrieval: retrieve candidate GO terms from proteins in the training data with similar descriptions 

Based on the assumption that similar proteins generally have similar GO functions, we develop our Retrieval component. As shown in Fig. 2b, given the features of target protein *x*, we retrieve a subset of candidate GO terms *G_r_*, from a subset *X_r_* (with top *k* most similar descriptions) of annotated proteins in the training data, where both $p_{\text{name}}$ and $p_{\text{species}}$ are used to measure the textual similarity. That is, for each protein *x_i_* in *X_r_*, we add the corresponding GO term set *G_i_* to the current set *G_r_*. Iterating this step, we obtain the set of candidates $G_{r}=\cup_{i=1}^{k}G_{i}$ and its definition $D_{r}=\cup_{i=1}^{k}D_{i}$. In order to reduce the time and space cost, we use a classical IR approach of BM25 (Lin *et al.* 2021) for measuring textual similarity.

### 2.4 Rerank: rerank retrieved GO terms using similarity between textual features and GO definitions

As shown in Fig. 2c, to calculate the relevance score between a target protein and retrieved GO terms, we consider a Cross-Encoder architecture, which is a BERT-based framework and computes interactions among tokens with self-attention mechanism (Reimers and Gurevych 2019). For each branch *b_i_*, given the features of a target protein x=(p,L) and one of the GO definitions *d_i_* from its corresponding retrieved set *D_r_*, the input of Cross-Encoder is defined as:


(1) $$\mathrm{Input}(x,d_{i},b_{i})=(<\text{CLS}>,\text{concat}(x,b_{i}),<\text{SEP}>,d_{i}),$$

and then we compute the semantic embedding of the input pair and apply a linear classifier to output the relevance score:


(2) $$E(x,d_{i},b_{i})={\text{BERT}}_{\text{CLS}}(\mathrm{Input}(x,d_{i},b_{i})),$$

(3) $$S(x,d_{i},b_{i})=\text{Sigmoid}(W^{T}*E(x,d_{i},b_{i})+b),$$

where ${\text{BERT}}_{\text{CLS}}$ denotes the embedding of the [CLS] token to express the interaction between $\text{concat}(x,b_{i})$ and *d_i_*. Also, to connect the protein description *p* and literature description *L*, we define the query $\text{concat}(x,b_{i})$ as:


(4) $$\begin{array}{c} \text{concat}(x,b_{i})=\text{concat}(p,L(b_{i}))\\ =<“\text{The protein is}”,p_{\text{name}},“\text{The description is}”,L(b_{i})> \end{array}$$

Here we choose the recommended names $p_{\text{name}}$ of a protein rather than the whole description *p*, since the species information is too shallow to capture functional similarity. $L(b_{i})$ is the extracted literature context of target protein *x* from the Sentence Extraction component.

### 2.5 Model training

Let *D*^+^ denotes the set of definitions of the relevant GO terms for target protein *x* and *D_r_* denotes the set of definitions of all candidate GO terms obtained in Retrieval, the negative sample set can be defined as D−={di−|di−∈Dr α di−∉D+}. Given a sample pair $\left(x,D^{+},D^{-}\right)$ for each branch *b_i_*, the loss function is:


(5) $$\begin{array}{c} L(x,D^{+},D^{-},b_{i})=-(\sum_{d^{+}\in D^{+}}\text{log}\;(S(x,d^{+},b_{i}))\\ +\sum_{d^{-}\in D^{-}}\;\text{log}\;(1-S(x,d^{-},b_{i}))), \end{array}$$

where S(·) is the score function given in (3) and $\text{concat}(x,b_{i})$ is the extracted query given in (4). To achieve better performance, we train a separate Cross-Encoder for each GO branch. Then the total loss for all proteins in training set $X_{\text{train}}$ to train the Cross-Encoder for a branch *b_i_* is defined as:


(6) $$L(X_{\text{train}},b_{i})=\sum_{x\in X_{\text{train}}}L(x,D^{+},D^{-},b_{i})$$

In training, we keep $|D^{+}|:|D^{-}|=1:1$ (where $\left|D^{+}\right|$ denotes the number of examples in *D*^+^) for optimal training results. So we randomly select $\left|D^{+}\right|$ negative samples from *D*^–.^

### 2.6 Alternative approaches for generating candidate GO terms

In Retrieval, candidate GO terms are generated based on the textual similarity using protein descriptions. It is worth noting that we can explore alternative approaches using other data sources rather than text to generate the candidate GO terms and investigate the possible enhancement of results by Rerank. In fact, in our experiments, we examine two alternative approaches for generating candidate GO terms: BLAST-KNN and LR-ESM, which are both two major component methods in NetGO3.0 (Wang *et al.* 2023).

## 3 Experimental setup

### 3.1 Datasets and evaluation metrics

We collected experimental annotations of proteins from SwissProt (Boutet *et al.* 2016), GOA (Huntley *et al.* 2015), and GO (Ashburner *et al.* 2000) in July 2023. Following the settings of CAFA5, we only consider functional annotations validated by experimental (or high-throughput) evidence (or traceable author statements) or inferred by curators (Friedberg *et al.* 2023).

The sequences of proteins with annotated GO terms are extracted from UniProt. We extract PubMed identifiers from each protein entry in SwissProt, following DeepText2GO (You *et al.* 2018a). Similarly, we obtain protein descriptions from SwissProt which include three parts: “*Protein names*,” an exhaustive list of all names of the corresponding protein; “*Gene names*,” the name list of genes that encode a protein; “*Organism names*,” showing the species information of each protein (The UniProt Consortium 2023). Besides, we downloaded the GO of 1 January 2023 and obtained definitions for every GO term.

To validate the effectiveness of GORetriever, following DeepText2GO, we randomly select 1000 SwissProt proteins for testing based on the species distribution of the CAFA5 test superset (around 140 000 proteins), and choose proteins that have literature information in SwissProt, resulting in three test datasets of 882, 861, and 811 proteins on MFO, BPO, and CCO, respectively. They are regarded as *text proteins*. The size of the test dataset is approximately 2 and 1.5 times that of NetGO2.0 and NetGO3.0, respectively, which were both demonstrated as a reliable performance indicator in previous CAFA challenges. Table 1 shows the statistics of datasets. Following the setting of CAFA5, we use the weighted maximum *F*-measure to evaluate the performance. It is calculated based on weighted precision and recall, in which the weights are the information content of the terms (Friedberg *et al.* 2023). The information content is defined as follows (Clark and Radivojac 2013):


(7) $$\text{IC}(v)={\;\text{log}\;}_{2}\frac{1}{\text{Pr}(v|Pa(v))},$$

where Pa(v) is the ancestor terms of term *v* and Pr(v|Pa(v)) denotes the conditional probability of term *v* given its ancestor terms in GO. Using the information content, we obtain the weighted *F*-measure as follows (Jiang *et al.* 2016):


(8) $${\text{wF}}_{\max}=\underset{\tau }{\max}\left\{\frac{2\cdot \text{wpr}(\tau )\cdot \text{wrc}(\tau )}{\text{wpr}(\tau )+\text{wrc}(\tau )}\right\},$$

where wpr(τ) and wrc(τ) are weighted-precision and weighted-recall under a threshold *τ*, given as follows:


$$\begin{array}{c} \text{wpr}(\tau )=\frac{1}{m(\tau )}\sum_{i=1}^{m(\tau )}\frac{\sum_{v}\text{IC}(f)\cdot 1(S(v,p_{i})\geq \tau )\cdot I(v,p_{i})}{\sum_{v}\text{IC}(v)\cdot 1(S(v,p_{i})\geq \tau )},\\ \text{wrc}(\tau )=\frac{1}{n_{e}}\sum_{i=1}^{n_{e}}\frac{\sum_{v}\text{IC}(f)\cdot 1(S(v,p_{i})\geq \tau )\cdot I(v,p_{i})}{\sum_{v}\text{IC}(v)\cdot I(v,p_{i})}, \end{array}$$

where m(τ) is the number of proteins with scores not smaller than *τ* for at least one GO term, *n_e_* is the number of test proteins, $S(v,p_{i})$ is the prediction score of protein *p_i_* on term *v* and $I(v,p_{i})$ denotes that protein *p_i_* has the function of term *v* (You *et al.* 2018b).

**Table 1.** The number of proteins of different species on three branches for training and testing.

	Train			Test		
	MFO	BPO	CCO	MFO	BPO	CCO
Human (*Homo sapiens*)	16 237	11 759	22 783	139	142	118
Mouse (*Mus musculus*)	9560	11 045	10 361	111	113	109
Drome (*Drosophila melanogaster*)	7134	10 985	9154	18	16	14
Arath (*Arabidopsis thaliana*)	8444	8756	8756	101	103	100
Rat (*Rattus norvegicus*)	5932	7000	5737	47	43	50
All species (not only the above)	79 866	90 014	95 988	882	861	811

### 3.2 Competing methods

We compare our method with important component methods in NetGO3.0: BLAST-KNN, LR-Text, LR-InterPro, LR-ESM (Wang *et al.* 2023). We also compare with two state-of-the-art sequence-based deep learning methods, DeepGOCNN (Kulmanov and Hoehndorf 2020), and ATGO (Zhu *et al.* 2022), which are re-trained on the same dataset of our method using their open sources. Note that all these competing methods are based on a single source of information. Furthermore, to explore the effect of incorporating protein descriptions, we add one more competing method, LR-ProtST, which uses ProtST to generate protein embedding from both protein sequences and descriptions.

**BLAST-KNN** aims to find similar proteins of a target protein and assigns annotated terms of the similar proteins to the target. We used BLAST to search similar proteins set *H_i_* for protein *p_i_* and the prediction score is computed as follows:


(9) $$S\left(v,p_{i}\right)=\frac{\sum_{p\in H_{i}}I\left(v,p\right)*B\left(p_{i},p\right)}{\sum_{p\in H_{i}}B\left(p_{i},p\right)},$$

where $B\left(p_{i},p\right)$ is the bit score (similarity) between two proteins, *p_i_* and *p*, from BLAST.

**LR-InterPro** uses protein families, domains, and motifs to generate a binary feature vector for each protein, which is then used to train logistic regression (LR) classifiers for each GO term (You *et al.* 2018b).

**Net-KNN** finds similar proteins from a PPI network and the prediction score is obtained as follows:


(10) $$S\left(v,p_{i}\right)=\frac{\sum_{p_{k}\in N_{i}}I\left(v,p_{k}\right)*\omega \left(p_{i},p_{k}\right)}{\sum_{p_{k}\in N_{i}}\omega \left(p_{i},p_{k}\right)},$$

where *N_i_* denotes the neighborhood of node *p_i_* in a PPI network (STRING) and $\omega \left(p_{i},p\right)$ is the weight of the association between *p_k_* and target protein *p_i_* (You *et al.* 2019).

**LR-Text** is the text component of DeepText2GO.

**LR-ESM** uses ESM-1b to generate protein embedding (Rives *et al.* 2021) and trains LR classifiers for each GO term (Wang *et al.* 2023). Then we predict protein functions based on the trained classifiers.

**LR-ProtST** uses ProtST to generate protein embedding and train LR classifier for each GO term.

**DeepGOCNN** uses 1D convolutional neural networks (CNNs) with different filter sizes to scan protein sequences and learns a feature vector with the size of 8192 for function prediction (Kulmanov and Hoehndorf 2020).

**ATGO** extracts functional features by a pretrained protein language model, ESM-1b, and implements high-precision function prediction with a triplet neural network (Zhu *et al.* 2022).

### 3.3 Model parameters setup

In Sentence Extraction, we choose the MonoT5 (Nogueira *et al.* 2020) model, which is trained based on MS MARCO corpus (Bajaj *et al.* 2018), as the Seq2seq backbone to compute semantic similarity between sentences from literature and protein descriptions. We sort all sentences and pick the top 50% as the informative sentences. In Retrieval, we choose the name and species information of proteins in UniProt to build the BM25 (Lin *et al.* 2021) index. In Rerank, for Cross-Encoder, we set the batch size as 8 and the warm-up ratio as 0.1. To embed the textual description, we use PubMedBERT (Gu *et al.* 2021), which is pre-trained by millions of biomedical papers to obtain state-of-the-art performance on specialized tasks. In training, we randomly select 10% of the data as the validation set. For Retrieval, we extract around 1000 proteins for each branch as the validation set, because obviously the more training data in Retrieval, the better results can be achieved. Another important parameter for Retrieval is the number of retrieved proteins *k*, that have similar descriptions as those of the target protein. Based on the F_1_ scores over GO terms retrieved by Retrieval, we set *k *=* *3 for MFO and BPO and *k *=* *2 for CCO that has less annotated GO terms (see Section 4.2 for more details).

## 4 Results and analysis

### 4.1 Comparison with competing methods over text proteins in the test set

The key idea of GORetriever is to annotate the functions of a protein with textual information. In the left part of Table 2, we report the results of GORetriever and the competing methods over *text proteins* in the test set. We have three main findings: (i) GORetriever achieves the best performance in all three branches, especially for BPO. For example, GORetriever achieves 7.1% and 12.1% improvements over the second and third-best methods, respectively. Moreover, GORetriever achieves the highest wF_max_ on average. This result demonstrates that GORetriever makes good use of textual information on proteins and GO terms in a reasonable and efficient way. (ii) Sequence-based methods are effective for MFO. BLAST-KNN (0.644) using sequence alignment performs better than LR-ESM (0.632) only utilizing ESM-1b. In contrast, LR-ProtST (0.649) using both ESM-1b and protein descriptions for embeddings achieves the second best. This suggests that protein embedding allows to capture functional information from amino acid sequences, and protein description is very helpful in generating better representation for AFP. (iii) Text information plays a significant role in predicting function on CCO. GORetriever, LR-ProtST and LR-Text, three text-related methods, outperform all sequence-based methods on CCO. This result indicates that the locations related to the cellular structures of proteins may have been annotated or implied in the scientific literature or protein descriptions.

**Table 2.** Performance comparison of competing methods.^a^

Method	Text proteins				Difficult proteins			
	MFO	BPO	CCO	Ave. wF_max_	MFO	BPO	CCO	Ave. wF_max_
	(882)	(861)	(811)		(404)	(419)	(362)	
*Sequence-based method*								
BLAST-KNN	0.644	0.471	0.595	0.570	0.603	0.454	0.513	0.523
LR-InterPro	0.627	0.465	0.591	0.561	0.615	0.450	0.525	0.530
LR-ESM	0.632	0.449	0.607	0.563	0.592	0.448	0.591	0.544
LR-ProtST^b^	0.649	0.462	0.625	0.579	0.610	0.461	0.613	0.561
DeepGOCNN	0.573	0.409	0.547	0.510	0.532	0.402	0.515	0.483
ATGO	0.642	0.509	0.589	0.580	0.611	0.487	0.599	0.566
*Network-based method*								
Net-KNN	0.400	0.458	0.596	0.485	0.422	0.490	0.611	0.508
*Text-based method*								
LR-Text	0.520	0.486	0.619	0.541	0.502	0.479	0.606	0.529
GORetriever	**0.659**	**0.545**	**0.653**	**0.619**	**0.619**	**0.573**	**0.651**	**0.614**

a The bold and underlined numbers denote the best and second-best performances, respectively. The figures in brackets denote the number of test proteins.

b LR-ProtST is also a text-related method, where ProtST uses both protein sequence and descriptions to generate embedding.

Furthermore, we focus on *difficult proteins* [called by CAFA (Zhou *et al.* 2019)] in the test set with the BLAST identity of <0.6 to any proteins in training data. In the right part of Table 2, we present the prediction performance of competing methods over *difficult proteins*. We have the following three observations: (i) GORetriever achieves the best performance in all three branches, indicating the robustness of GORetriever over *difficult proteins*. (ii) LR-ProtST outperforms LR-ESM again in all three branches. This is a strong indication of the effectiveness of incorporating protein descriptions into protein embedding for better AFP. (iii) Most sequence-based methods show heavy performance decreases, especially BLAST-KNN. BLAST-KNN achieves an average wF_max_ of only 0.523, suggesting that their performances heavily rely on homologous proteins.

All these results demonstrate that GORetriever is the most effective and robust among all competitive methods over *text proteins* and especially *difficult proteins*. We also present the prediction performances by other metrics (*S_min_*) in the supplement, where GORetriever also achieves the highest performances.

### 4.2 Robustness analysis

**Figure 3.**
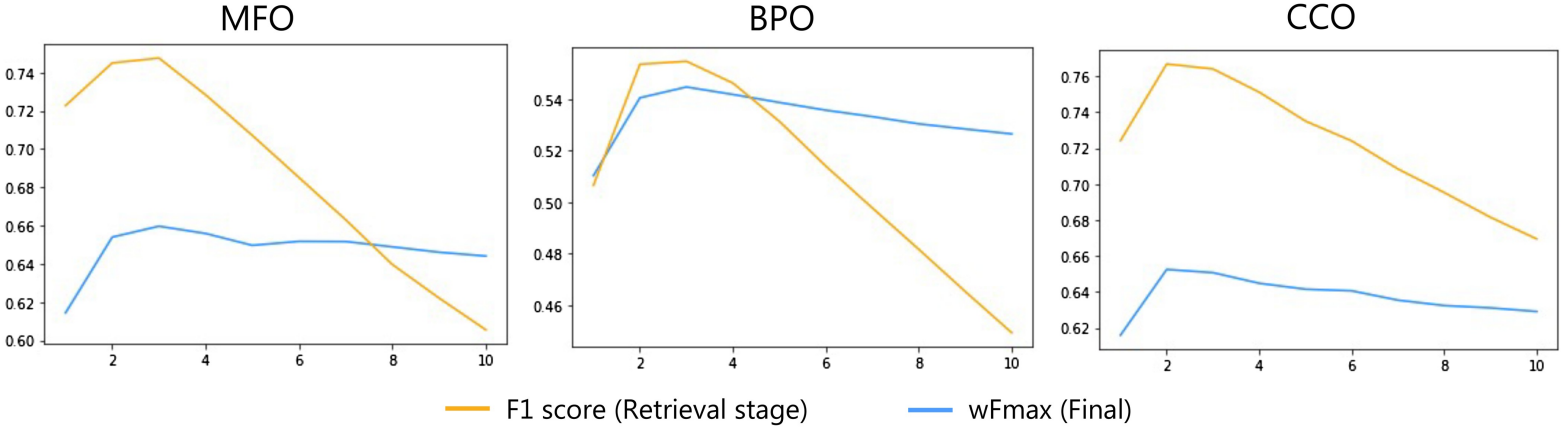
F_1_ score and wF_max_ scored by Retrieval only and GORetriever, respectively, changing *k*.

To determine the value of top *k*, we evaluate the F_1_ score (a standard performance measure in information retrieval) of Retrieval on the validation set. Note that the F_1_ score allows us to balance accuracy and cost. Figure 3 shows the F_1_ scores on the validation set, first increasing and then decreasing with increasing *k*, as a consistent trend over all three sets. Thus, we adopt *k *=* *2 for CCO and *k *=* *3 for MFO and BPO as the parameter values. We compute wF_max_ on the test set with different *k* values to explore the robustness of GORetriever. Figure 3 shows the wF_max_ curves, implying that (i) the trends of wF_max_ and F_1_ scores are consistent, suggesting that choosing the optimum parameter values of *k* would be possible, implying that similar proteins have similar functions, and (ii) the change of wF_max_ (when changing *k*) is smoother than F_1_ scores. We believe that Rerank in the second stage could further improve the annotation ability of GORetriever and more importantly, make the model, GORetriever, more robust against the change of parameter *k*.

### 4.3 Ablation experiment

*GORetriever is improved progressively during two stages.* We conducted ablation experiments to understand the contribution of each component in GORetriever. Given a target protein *x*, for GO term *g_i_* associated with relevant proteins in Retrieval, we compute the score *S* between *g_i_* and *x*, following the idea of BLAST-KNN (You *et al.* 2018b):


(11) $$S(g_{i},x)=\frac{\sum_{x^{\prime }\in X_{r}}I(g_{i},x^{\prime })*B(x,x^{\prime })}{\sum_{x^{\prime }\in X_{r}}B(x,x^{\prime })},$$

where $B(x,x^{\prime })$ stands for the relevance score obtained by the BM25 algorithm, and $I(g_{i},x^{\prime })$ is an indicator whether protein $x^{\prime }$ has the function of *g_i_*. We use the same setting of *k* as discussed in Section 4.2. Table 3 shows the results, implying that (i) both components (Retrieval and Rerank) are beneficial to the result. Using Retrieval only has achieved better performances than LR-ESM (4.8%) and BLAST-KNN (3.5%), and (ii) Adding Rerank improves the annotation ability for *difficult proteins*. The improvement from Retrieval by Rerank is 4.9% on *text proteins*, but this improvement becomes 6.8% on *difficult proteins*.

**Table 3.** Ablations of using Retrieval or sequence-based AFP methods and then Rerank.^a^

Method	Text proteins				Difficult proteins			
	MFO	BPO	CCO	Ave. wF_max_	MFO	BPO	CCO	Ave. wF_max_
	(882)	(861)	(811)		(404)	(419)	(362)	
Retrieval	0.621	0.519	0.629	0.590	0.570	0.535	0.619	0.575
+Rerank	0.659	0.545	0.653	0.619	0.619	0.573	0.651	0.614
LR-ESM	0.632	0.449	0.607	0.563	0.592	0.448	0.591	0.544
+Rerank	0.628	0.496	0.615	0.579	0.608	0.510	0.614	0.577
BLAST-KNN	0.644	0.471	0.595	0.570	0.603	0.454	0.513	0.523
+Rerank	0.633	0.495	0.598	0.575	0.605	0.480	0.566	0.550

a A denser shadow means more performance improvement.

*GORetriever enhances the performance of sequence-based methods via Rerank.* We choose the top 100 GO terms from the predictions of each of GORetriever, LR-ESM and BLAST-KNN as the input of Rerank. Table 3 shows the results (wF_max_), implying that (i) GORetriever yields different levels of enhancement for sequence-based methods by Rerank. The improvement is especially significant on BPO, which has more GO terms with deeper parts in the ontology structure, making prediction more challenging for traditional classification methods, and (ii) GORetriever shows robustness: Compared to *text proteins*, the results on *difficult proteins* are reduced for all methods, but the performance reduction is significantly alleviated by using Rerank.

### 4.4 Performance over CAFA5 blind test set

**Figure 4.**
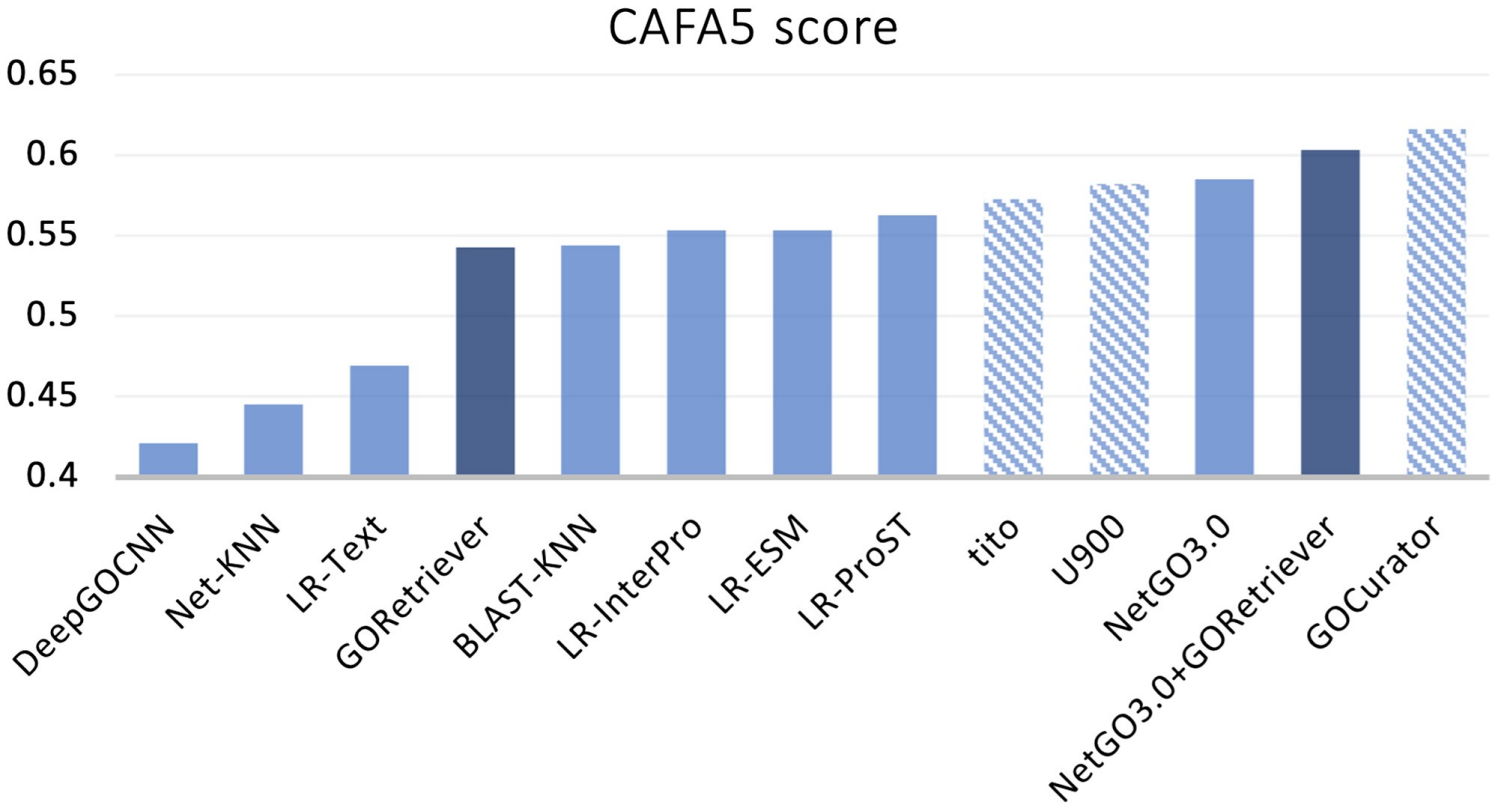
Results over the CAFA5 blind test set. The top three methods (using diagonal lines) in CAFA5: GOCurator, U900, and tito (both U900 and tito are sequence-based), are added. Our methods are indicated by the dark color.

In CAFA5, the organizers provided a large number of protein sequences (test superset), which consists of around 140 000 proteins, where around 92% of these proteins have annotated literature. Participants of CAFA5 made their predictions over all proteins in the test superset, and then wF_max_ was used to rank the submissions of all participants over a blind test set (an unknown number of proteins out of test superset). Following this protocol, we examine the performance of GORetriever and the competing methods over the CAFA5 blind test set. Figure 4 shows the performance results of all methods as well as, additionally, the top three submissions in CAFA5 (GOCurator, U900 and tito) for reference. The best method is GOCurator, an ensemble method (based on NetGO3.0), allowing to integrate protein structure-, sequence-, and text-based methods, including BLAST-KNN, LR-ESM, and so on. Note that we have proposed GOCurator for CAFA5, and GORetriever was the new and key component of GOCurator. From this result, we have the following three observations: (i) GORetriever outperformes LR-Text significantly, highlighting the methodological advantage of GORetriever over LR-Text. (ii) Incorporating GORetriever into NetGO3.0 successfully improves the wF_max_ performance (from 0.587 to 0.604). This demonstrates that the strategy of GORetriever for predicting GO functions based on literature annotations is robust and can complement traditional AFP methods (which were used as other components in NetGO3.0). (iii) Without any knowledge of the proportion of text proteins in the blind test set, GORetriever achieves a competitive performance against BLAST-KNN, LR-ESM, and LR-InterPro, which all do not use any text information. This is consistent with the results of all test proteins (including both text and nontext proteins) in this study (see Supplementary Material).

### 4.5 Case study of protein O82234

Table 4 shows the prediction results of GORetriever and six typical competing methods for protein O82234 (“Translation initiation factor IF3-2”) in BPO (results of all 11 competing methods are shown in Supplementary Table S3). Methods based on protein embedding information perform unfavorably, such as LR-ESM, which could predict only one correct term (F_1_ score of 0.076). ATGO using ESM-1b (with a triple neural network) outperforms LR-ESM with an F_1_ score of 0.357. In contrast, GORetriever based on textual information achieves the best F_1_ score of 0.667 out of all 12 methods, and LR-text obtains a favorable F_1_ score of 0.47. Enhancing the base model by Rerank using textual information is also shown: BLAST-Rerank (0.296) and ESM-Rerank (0.545) yield significant improvements over BLAST-KNN (0.250) and LR-ESM (0.076), respectively. However, BLAST-Rerank and ESM-Rerank could not perform at the same level as GORetriever (0.667), due to the unsatisfactory performances of base models, i.e. BLAST and ESM. This result highlights the importance of generating good candidate GO terms in the first stage.

**Table 4.** Prediction results for O82234 in BPO.^a^

Method	TP	FP	F_1_-score
BLAST-KNN	4	14	0.250
LR-Text	8	12	0.471
LR-ESM	1	11	0.076
ATGO	5	9	0.357
BLAST-Rerank	4	9	0.296
ESM-Rerank	9	10	0.545
GORetriever	11	8	**0.667**

a TP and FP are the numbers of true and false positives out of all 16 GO terms. The bold number denote the best performance. The root term “biological process” (GO:0008150) is excluded. Detailed results of all 12 methods are shown in the Supplementary material.

To further investigate the impact of using literature annotation, we focus on two GO terms “chloroplast organization” (GO:0009658) and “system development” (GO:0048731), which are both correctly predicted only by GORetriever or its variants (e.g. ESM-Rerank). Table 5 shows sentences (relevant to these two GO terms), which are extracted based on computations by Rerank. We can see that GORetriever effectively captures the semantic correlation between extracted sentences and GO definitions, such as “disassembly of the chloroplast” and “chloroplast development,” resulting in facilitating the annotation of GO functions. Supplementary Fig. S2 illustrates the ontology graph based on the relations among 16 GO terms that are annotated to O82234 in BPO. Compared to the competing methods, both the text-based LR-Text and GORetriever correctly predict the “developmental process” branches. This result clearly demonstrates the advantage of incorporating textual information for AFP.

**Table 5.** GO term, GO definition and predicted relevant sentences of O82234: translation initiation factor IF3-2 in BPO.

GO term	GO definition	Relevant Sentences
chloroplast organization (GO:0009658)	A process that is carried out at the cellular level which results in the assembly, arrangement of constituent parts, or **disassembly of the chloroplast**.	Genetic and molecular evidence indicate that SVR9 and its close homolog SVR9-LIKE1 (SVR9L1) are functionally interchangeable and their combined activities are essential for **chloroplast development** and plant survival.
system development (GO:0048731)	The process whose specific outcome is **the progression of an organismal system over time**, from its formation to the mature structure. A system is a regularly interacting or interdependent group of organs or tissues that work together to carry out a given biological process.	Interestingly, we found that SVR9 and SVR9L1 are also involved in **normal leaf development**. Genetic analysis established that SVR9/SVR9L1-mediated **leaf margin development** is dependent on CUP-SHAPED COTYLEDON2 activities and is independent of their roles in **chloroplast development.**

Bolded text represents evidence that can support the final function annotation.

To further elucidate the influence of textual information on predictive outcomes, we focus on the “false positive” GO categories identified by GORetriever. Notably, several of these categories are broad and general in nature, such as GO:0065007 (biological regulation), GO:0043170 (macromolecule metabolic process), and GO:0044238 (primary metabolic process). Although these categories may not precisely correspond to the target protein, the associated textual descriptions are sufficiently broad and ambiguous, thereby achieving a semantic match that may still capture some aspects of the protein’s biological functions. These results also suggest that integrating GORetriever with other models could yield unexpectedly nuanced outcomes.

## 5 Conclusion

We have presented GORetriever for accurate automated function annotation of proteins. For a given target protein, GORetriever first generates GO term candidates using protein descriptions and also relevant informative sentences (from literature), and then reranks the GO terms by deep information retrieval on these informative sentences. Through extensive experiments on *text proteins*, we demonstrate that GORetriever can annotate protein functions accurately by using textual information, especially powerful for predicting *difficult proteins*. Furthermore, we replace the Retrieval of GORetriever with existing sequence-based AFP methods and find that the performance of these methods can be always enhanced by Rerank of GORetriever, indicating that Rerank is universally effective for AFP. Possible future work would be to create an approach to incorporate the interactions between/among GO terms to improve the current AFP performance further.

## Supplementary Material

btae401_Supplementary_Data

## Data Availability

The data is available at https://github.com/ZhuLab-Fudan/GORetriever/tree/main/data.
