# Supplementary figures and images for: GORetriever: reranking protein-description-based GO candidates by literature-driven deep information retrieval for protein function annotation
Source: Bioinformatics. 2024 Sep 4;40(Suppl 2):ii53–61. doi: 10.1093/bioinformatics/btae401 (PMC11520413; doi:10.1093/bioinformatics/btae401)

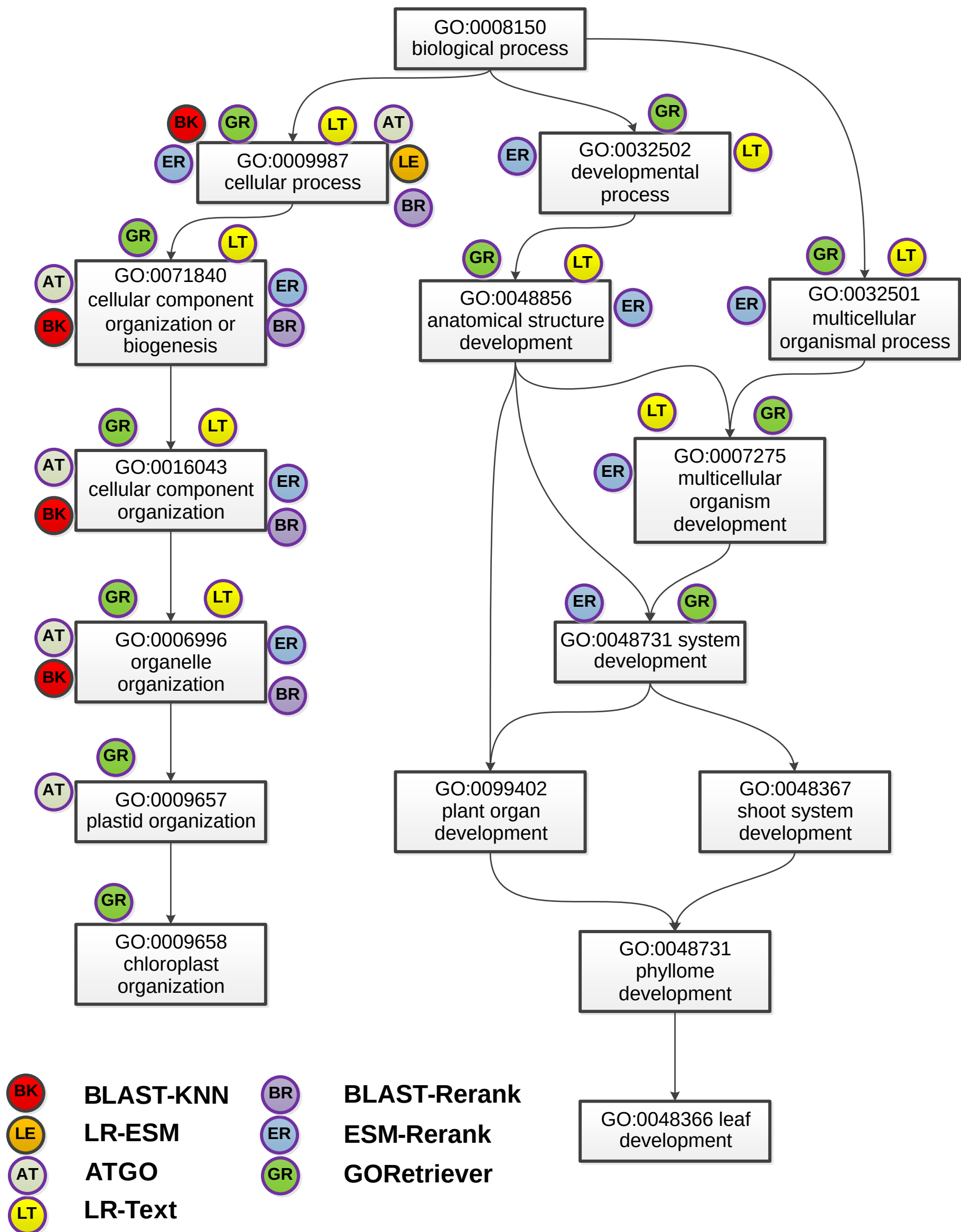

Supplement: btae401_Supplementary_Data [file btae401_supplementary_data.zip › case.pdf]

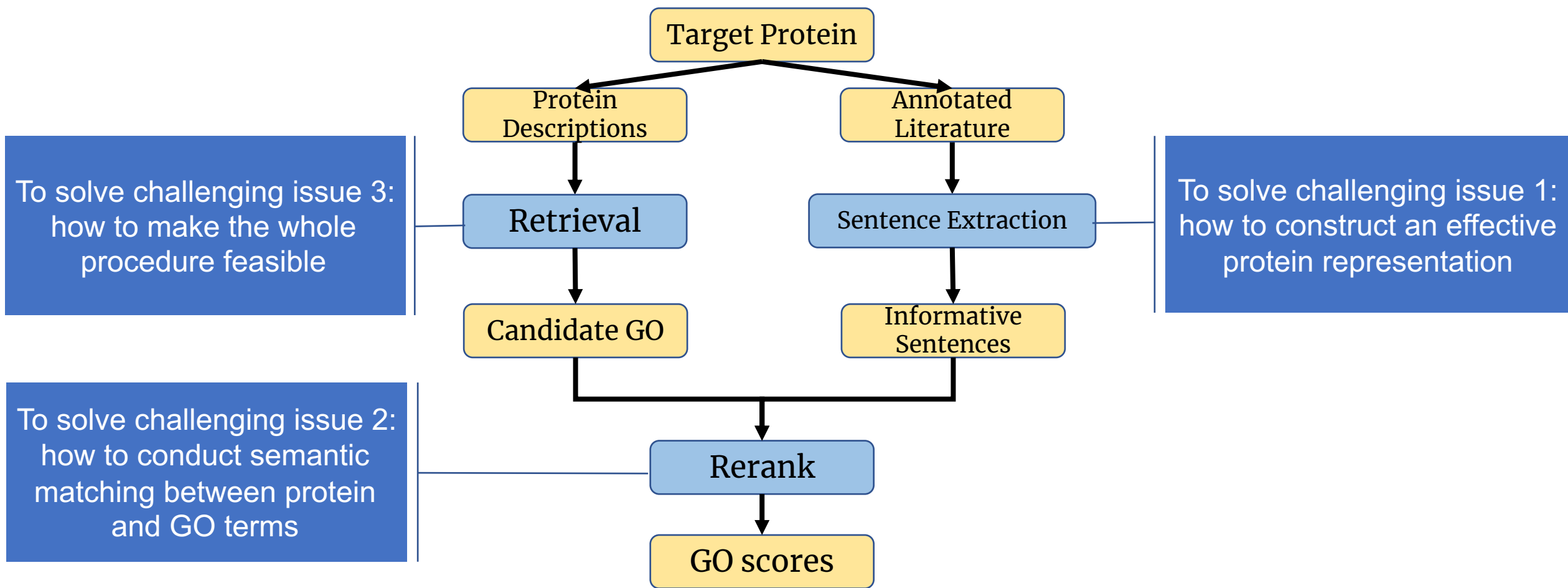

Supplement: btae401_Supplementary_Data [file btae401_supplementary_data.zip › framework.pdf]

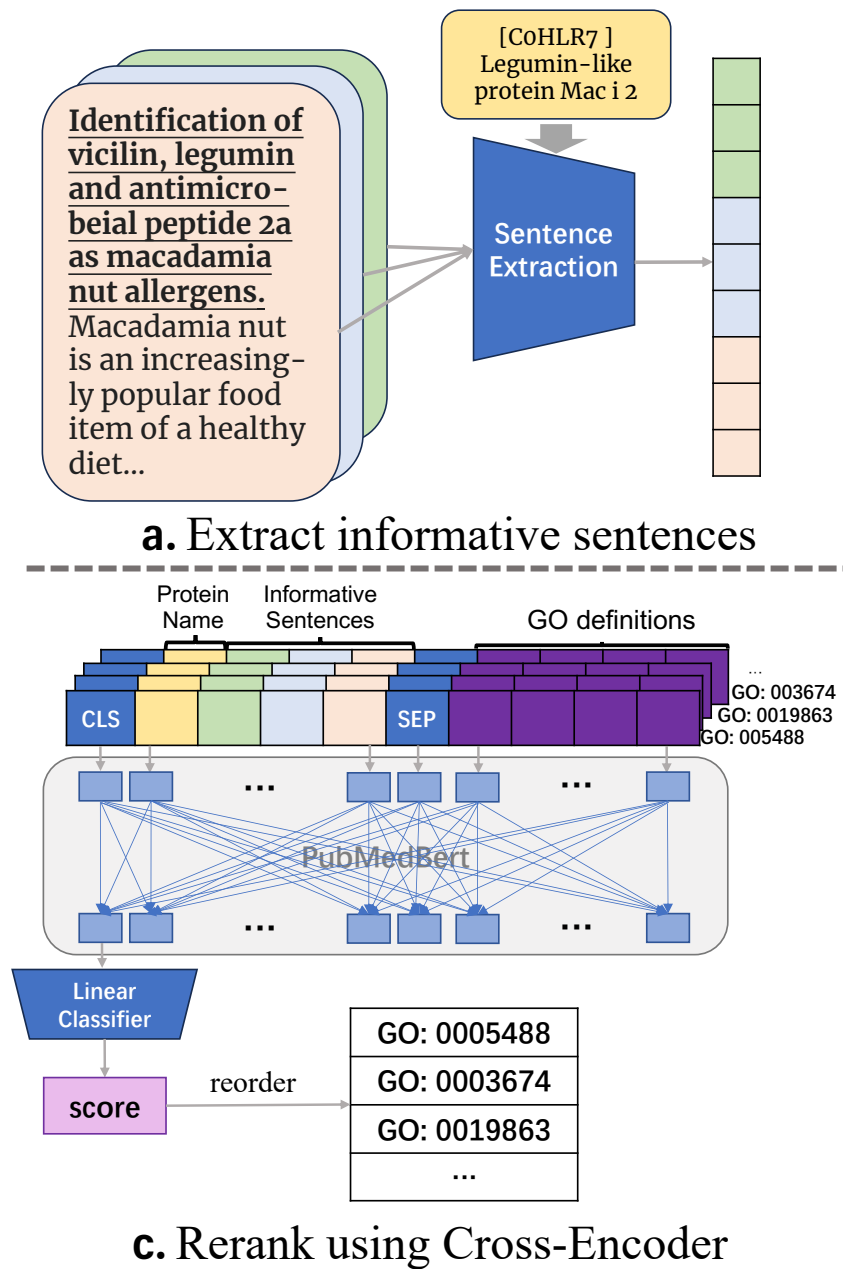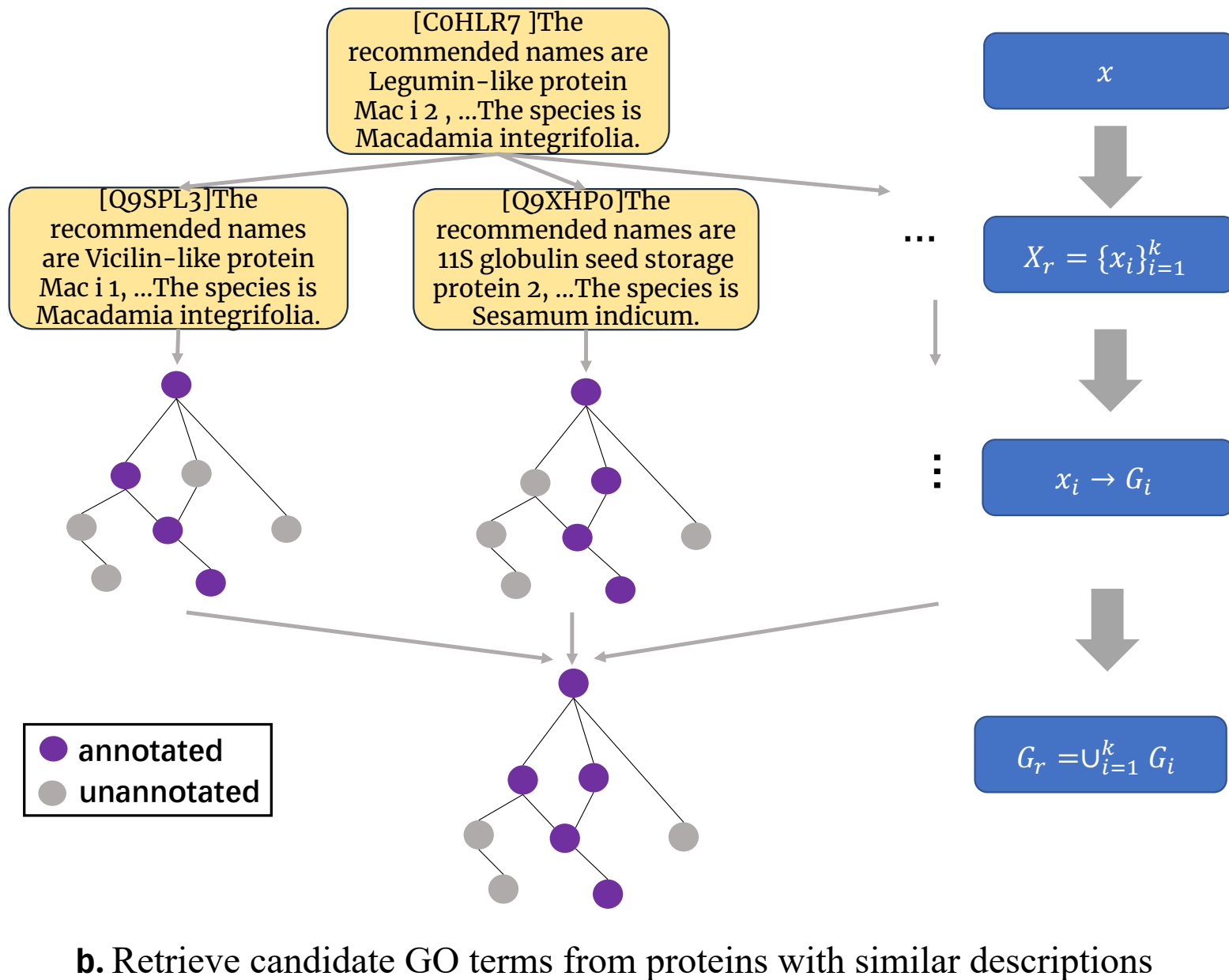

Supplement: btae401_Supplementary_Data [file btae401_supplementary_data.zip › process.pdf]

MFO

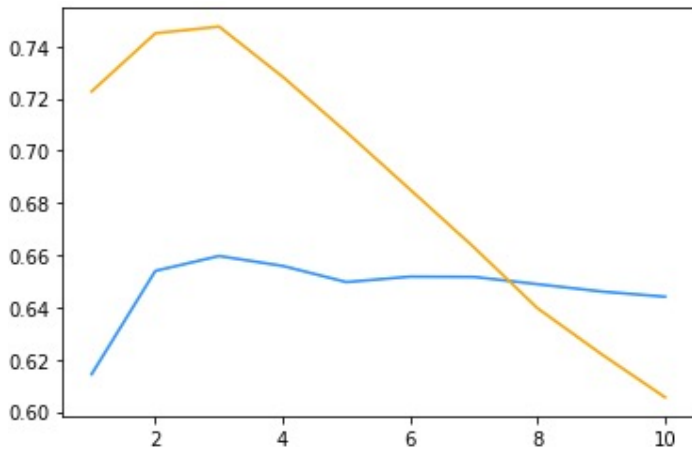

BPO

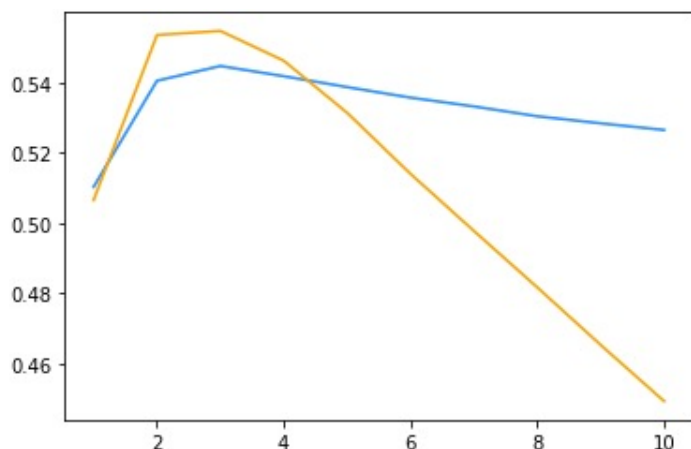

CCO

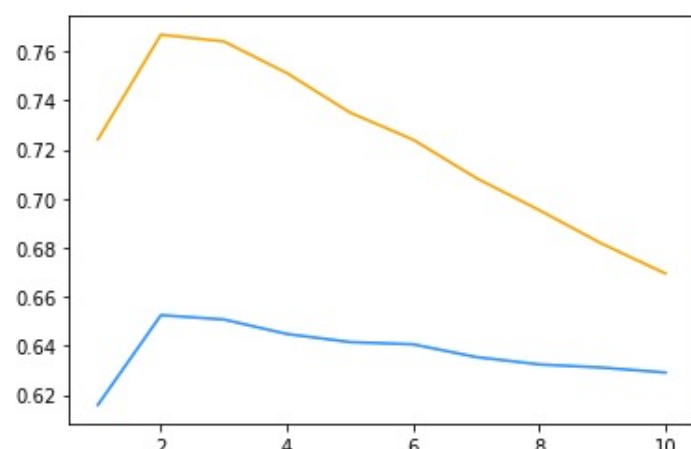

— F1 score (Retrieval stage)

— wFmax (Final)

Supplement: btae401_Supplementary_Data [file btae401_supplementary_data.zip › select.pdf]
